# In silico optimization for production of biomass and biofuel feedstocks from microalgae

**DOI:** 10.1007/s10811-014-0342-2

**Published:** 2014-06-01

**Authors:** Philip Kenny, Kevin J. Flynn

**Affiliations:** Centre for Sustainable Aquatic Research, Department of Biosciences, College of Science, Swansea University, Singleton Park, Swansea, SA2 8PP UK

**Keywords:** Microalgae, Biomass, Biofuel, Optimisation, Nutrients

## Abstract

Optimization of the production rate of biomass rich in N (e.g. for protein) or C (e.g. for biofuels) is key to making algae-based technology commercially viable. Creating the appropriate conditions to achieve this is a challenge; operational permutations are extensive, while geographical variations localise effective methods of cultivation when utilising natural illumination. As an aid to identifying suitable operational envelopes, a mechanistic acclimative model of microalgae growth is used for the first time to simulate production in virtual systems over a broad latitudinal range. Optimization of production is achieved through selection of strain characteristics, system optical depth, nutrient supply, and dilution regimes for different geographic and seasonal illumination profiles. Results reveal contrasting requirements for optimising biomass vs biofuels production. Trade-offs between maximising areal and volumetric production while conserving resources, plus hydrodynamic limits on reactor design, lead to quantifiable constraints for optimal operational permutations. Simulations show how selection of strains with a high maximum growth rate, *U*_*m*_, remains the prime factor enabling high productivity. Use of an f/2 growth medium with a culture dilution rate set at ~25 % of *U*_*m*_ delivers sufficient nutrition for optimal biomass production. Further, sensitivity to the balance between areal and volumetric productivity leads to a well-defined critical depth at ~0.1 m at which areal biofuel production peaks with use of a low concentration f/4 growth medium combined with a dilution rate ~15 % of *U*_*m*_. Such analyses, and developments thereof, will aid in developing a decision support tool to enable more productive methods of cultivation.

**Electronic supplementary material:**

The online version of this article (doi:10.1007/s10811-014-0342-2) contains supplementary material, which is available to authorized users.

## Introduction

Major hurdles to realising the potential of algal biomass as a source of sustainable feed for food production, biotechnology, and ‘green’ biofuel include having the ability to produce and extract the required biochemicals cheaply, efficiently and on an industrial scale (Greenwell et al. 2010). Of these, optimization of biomass production is widely held as a, if not the, critical step in the exploitation of high value products (Stephens et al. 2010; Borowitzka 2013). In contrast, the emphasis in studies of algal biotechnology has tended to be on increasing the relative content of the target biochemical. In reality, it is the optimisation of the production rate of those specific biochemical components per unit of effort, set against their market value, which is the critical issue.

Rather less attention appears to have been given in publications to manipulating cell composition primarily for the purpose of optimising production of biofuels or as feed in intensive aquaculture, either via modification of growth conditions and/or of the genetics of the organisms themselves through GM. While the use of GM, transgenics and similar approaches have clear potential, concerns may be raised over various environmental risks (Flynn et al. 2010a, 2013). A more cautious approach is to make the most of wild strains, modifying growth conditions in order to maximise production of the required components. The other dichotomy in approaches is the use of natural versus artificial illumination. For high quality, high biosecurity and high repeatability of production, artificially lit enclosed bioreactors offer clear advantages. However, for truly intensive production, natural illumination is required else the financial if not the energetic costs of artificial lighting become prohibitive. Likewise, for local low-technology solutions, the use of solar illumination is most likely the favoured first option.

In this paper, the term ‘biofuel’ refers to any form of excess carbon stored by the cell and surplus to its immediate requirements which can be extracted and exploited for use in fuel technology. Such energy-rich substances include fatty acids and other lipids for use in biodiesels plus carbohydrates for bioethanol (Fon Sing et al. 2013). It should be noted though, that (depending on the source organism) fatty acids are also potentially important dietary feedstocks, especially as polyunsaturated fatty acids (the conflict being that bioenergy favours short chain saturated fatty acids). The contrary use of this algal biomass is for protein or high value compounds such as photo-protection pigments.

While undoubtedly, a biorefinery approach (Greenwell et al. 2010) gives maximum flexibility, ultimately whether a particular batch of product is intended to be C- or protein-rich presents a basic division point in the process. Finding the ideal combination of light and nutrient availability to encourage production of excess carbon versus protein-rich biomass is not a trivial exercise; optimising for biofuel production does not follow directly as a result of optimising biomass production. This is because maximum growth and enhanced lipid production tend to be mutually exclusive (Flynn et al. 2010a; Scott et al. 2010). Lipids accumulate when algae are N stressed (Flynn et al. 1993), a condition that is contrary for rapid biomass growth but one that can be exploited when developing strategies to maximise yields of oils and fatty acids by manipulating cell physiology (Li et al. 2008; Beer et al. 2009; Rodolfi et al. 2009; Greenwell et al. 2010). Optimal conditions may be achieved through selection of dilution rates in continuous culture systems to balance growth and N limitation, or in batch culture systems through a two-stage process whereby biomass is allowed to grow optimally and is subsequently N starved. However, there is the added complication that a dense N-starved culture is typically too self-shaded to allow sufficient light penetration to maximise the lipid yield within a reasonable time frame, if at all (Flynn et al. 2010a; Su et al. 2011).

The fundamental requirement for high levels of irradiance to enable rapid biomass and then, as applicable, excess C production, is of particular concern for growth using natural illumination at higher latitudes. Lower solar elevation and shorter winter days, combined with increased average cloud cover, decrease the amount of light available compared to locations at equatorial and intermediate latitudes. Requirements for artificial light to augment natural illumination need to be kept to a minimum to decrease costs. Thus, if one is to attempt cultivating algae on a commercial scale in, for example, northwest Europe, using methods found effective in the Southern USA or Australia, the first step should be to adjust expectation of yields to match local light availability, and modify operations accordingly.

To date, estimates of production at a given location have typically been made by calculating (with varying degrees of sophistication) photosynthetic activity based on local irradiance profiles and presumed algal photo-efficiency. However, the resulting broad range of projections has produced much uncertainty as to what is realistically achievable (Williams and Laurens 2010; Ritchie and Larkum 2012). What such calculations lack is the ability to adequately capture the physiological response of cells to the interplay between external environmental factors; for instance, to the shifting balance between light and N limitation, described above, which has so much bearing on lipid production rates. A more effective way of simulating these dynamics is by employing mechanistic acclimative models of algal growth.

As an aid to developing improved cultivation methods, mathematical models of algal growth can be utilised to build and run virtual systems simulated *in silico*, providing theoretical projections of possible yields. Such computer-based experiments can be performed in only a fraction of the time and cost of the ‘real world’ equivalent. The model on which this investigation is based (Flynn 2001) is an acclimative mechanistic model that is able to capture the dynamics of multi-nutrient interactions and can be modified to function within a wide range of virtual environments. While models exist for simulating detailed aspects of algal physiology (Novoderezhkin and van Grondelle 2010; Gorbunov et al. 2011; Papadakis et al. 2012; Xin et al. 2013) the critical issue here is relating biomass production to nutrients and light. This requires an intermediate level of description, between the extreme simplicity of models typically used in earlier analyses (Weyer et al. 2010) and systems biology approaches that cannot yet capture growth dynamics (not least because we lack the data sets to test such models). Features of the model used here include potential for a full representation of variable elemental stoichiometry (C, N, P, Fe and, for diatoms, Si) driven by variable temperature, nutrient (NH_4_^+^, NO_3_^−^, PO_4_^−^, bioavailable Fe, and SiO_4_) and light regimes (describing variable Chl:C with photoacclimation and nutrient status). Its effectiveness has been demonstrated repeatedly against data for various species, for diverse aspects of growth under varying conditions including (but not limited to) biomass and photoacclimation (Flynn et al. 2001; John and Flynn 2002), nutrient quotas and transport controls (Flynn 2008), production of dissolve organic matter (Flynn et al. 2008) and applications to ecology (Fasham et al. 2006; Flynn 2010). The production of excess C (carbohydrate and fatty acids) can also be simulated (Flynn et al. 2013).

For the first time, this model is applied to an investigation into the effects of multi-nutrient interactions on potential algae production using solar irradiance over a broad range of latitudes over the Earth’s surface. The projected geographical and seasonal variations in both biomass and biofuel (excess C) production are explored under a range of operational conditions, providing a framework for future more detailed and empirical studies, as well as sensitivity and financial analyses. What we present is thus a best case scenario, assuming no downtime for maintenance or system failures and also that certain engineering challenges are met.

## Methods

Production of biomass and biofuel from microalgae processing varied strain characteristics was simulated in different operational scenarios over a range of latitudes. Values for model parameters are presented in Table S1 in the Supporting Material. The model structure can be operated with variable CO_2_/pH and temperature, and with variable culture manipulations (semi-continuous etc.), though for reasons of brevity, here, we have made some simplifying assumptions. While there are undoubted challenges in the use of continuous culture techniques, there are at least as many for discontinuous culture approaches (see ‘Discussion’ section). Accordingly, and consistent with traditional microbial biotechnology approaches, we have simulated chemostat-style continuous culture. The following variables were explored.

### Latitude.

The latitudes considered range from 0° to 65° at 5° intervals, enabling a consideration of the latitudinal dependent effect of geographical and seasonal variations in natural light availability upon production rates. Latitude informed a solar cycle function to simulate diurnal and seasonal variations in available natural light:

1 $$ PFD= SC\left({ \cos}^{-1}\left[ \sin \varphi \kern0.5em  \sin \delta - \cos \varphi \kern0.5em  \cos \delta \kern0.5em  \cos \theta \right]\right) $$

where *SC* stands for the solar constant in micromole photons per square meter per second, φ is the latitude in radians, δ is the solar declination angle and θ is the angular description of the diel solar cycle. This expression is supplemented by atmospheric data (eosweb 2012) providing an average insolation clearness index between 0 and 1 for each latitude (typically, it varies between 0.45 and 0.7). The clearness index provides a means of estimating the fraction of sunlight penetrating the atmosphere on an average day (accounting for cloud cover, dust, etc.). Multiplying Eq. (1) by the latitude dependent clearness index thus adds further geographical variation. Although algae only utilise a fraction of the available PFD for photosynthesis (Ritchie 2010), the model is parameterized to account for this.

### Operational parameters.

Each virtual system was assumed to be a flat incubator, and optimally regulated with respect to temperature, CO_2_ supply and pH (pH increases with photosynthesis as CO_2_ is fixed). For large culture facilities, CO_2_ is likely to be obtained from adjacent (exothermic) industrial activity. As such, the energy for thermal regulation and the CO_2_ to support algal growth may be expected to be available, with CO_2_ introduced as part of a pH-stat control mechanism.

A number of operational parameters were varied to optimise production from each given system. The dilution rate was varied between 0 and 1 system volumes per day. Nutrient concentration levels were chosen to be 0.5×, 1.0×, 1.5×, and 2.0× the classic f/2 medium (Guillard and Ryther 1962; Guillard 1975), holding the N:P ratio constant throughout. (The f/2 medium contains 12.35 mg N L^−1^ and 1.11 mg P L^−1^.) Because of the form of the relationship between the algal N:C and P:C cell quotas and nutrient-limited growth rate (Flynn 2008), such media effectively drive N-limited growth under suitable illumination. Optical depths were considered over the range 0.03 ≤ *τ* ≤ 0.5 m, and irradiance refers to photosynthetically active radiation (PAR). Total (depth integrated) photosynthetic activity, PS, in the water column was calculated by analytically integrating the Smith equation (Smith 1936; Fasham et al. 2006) over the optical depth τ, assuming a homogeneous cell suspension, and by reference to the current value of algal Chl:C (i.e. to the amount of pigment in the algae which itself varies with photoacclimation and nutrient status). While the Smith equation can have a tendency to overestimate PS because of its inability to capture surface photodamage effects (Ritchie 2010), this choice of equation is made because it yields an analytical solution which provides a computationally cheap means to describe photosynthesis with sufficient rigour:

2 $$ PS=\frac{ Pqm}{ k\tau}\left[ \ln \left(\frac{I_o\alpha ChlC}{ Pqm}+\sqrt{1+{\left(\frac{I_o\alpha ChlC}{ Pqm}\right)}^2}\right)- \ln \left(\frac{I_o\alpha ChlC}{ Pqm}{e}^{- k\tau}+\sqrt{1+{\left(\frac{I_o\alpha ChlC}{ Pqm}{e}^{- k\tau}\right)}^2}\right)\right] $$

where α is the photosynthetic efficiency at *I* = 0, *I*_0_ is the surface irradiance, *ChlC* is the ratio of chlorophyll to carbon, *Pqm* is the absolute maximum rate of photosynthesis and *k* is the attenuation factor of the culture and a function of ChlC. The last four are dynamic variables so their values are updated at each timestep to capture photoacclimation effects (see Flynn (2001) for further details of how they are calculated).

### Algal physiology.

The description of the model for algal physiology was that of Flynn (2001); Flynn (2003) and (2006) give further information and explanation of model structure and rational. This model gives a variable stoichiometric description of C:N:P:Chl within an acclimative framework. Thus, with decreased light availability Chl:C increases until a maximum is attained, and decreases under nutrient stress and/or increased light availability, while nutrient transport is controlled externally by availability and internally by feedbacks from satiation. The model has been used widely over the last decade, fitted to data from many algae types, with examples referenced in the ‘Introduction’ section.

There are fundamental differences in microalgae across different taxa especially with respect to their minimum N:C and P:C quotas (Geider and LaRoche 2002). These, and especially N:C, impact upon their capacity to accumulate excess C, with maximum scope requiring a large difference between maximum N:C (NC_max_; needed to enable maximum growth) and minimum N:C (NC_min_; zero growth, with maximum excess C content); see Flynn et al. (2013) for further details. Here, these minimum quota values were held at the lower end of the spectrum (enabling a high biofuels production under optimal conditions). Likewise, minimising the level of photoacclimation has an important impact on the potential for self-shading (Flynn et al. 2010a, 2013); here, a typical level of pigmentation was assumed (0.06 gChl gC^−1^; i.e. with no GM or other selection for a lower maximum Chl:C). This leaves the maximum growth rate *U*_*m*_ as the most important feature of algal growth affecting production (see Flynn et al. (2013) for further information and justification). Maximum growth rate was considered over the range of 0.5 to 2 doublings per day; a doubling per day equates to a growth rate of 0.693 day^−1^. (It should be noted that the value of *U*_*m*_ describes the maximum growth rate achievable under continuous illumination, while the simulations were conducted under the diel light cycle, changing each day over the year for most latitudes.) Different physiological configurations (different growth rates) are hereafter attributed to different ‘strains’. The majority of parameters describing the physiology remained common to each strain and were set according to those tabulated for the non-GM model described by Flynn et al. (2013), the exception being maximum growth rate *U*_*m*_ (see Table S1 in the Supporting Material). While these values are typical of those measured experimentally, rather than strain specific (Flynn 2001), the chosen maximum growth rates equate to those of strains considered for commercial production; for instance a choice of *U*_*m*_ = 0.693 day^−1^ would be relevant to cultivation of strains of *Nannochloropsis* (Boussiba et al. 1987; Flynn et al. 2010b) and *Spirulina* (Lee 2010) while a choice of *U*_*m*_ = 1.386 day^−1^ would be more relevant to cultivation of *Scenedesmus* (Lee 2010) and diatoms (Lourenco et al. 2002).

### Modelling strategy and conventions for presentation of results.

The model was operated within the Powersim Constructor v2.51 platform (Isdalstø, Norway). In order to guide subsequent investigations, scoping simulations were run to ascertain the optimal dilution rates required to cultivate each strain at an intermediate latitude. This was done using Powersim Solver v.2, to maximise yearly production in terms of biomass and also of excess C (hereafter termed, Cex_C_). Solver populates an array with possible initial parameters and automates the solution search by means of an evolutionary (‘genetic’) algorithm to find the optimal configuration to maximise output. Once optimal solutions were found, the surrounding solution space was explored manually to investigate the effects of altering model parameters.

Efficient use of space, water, nutrients and energy in a commercial facility requires an optimal balance of areal and volumetric production. Currently, open-pond systems remain the only viable option for industrial-scale cultivation of algae for biofuel feedstocks (Greenwell et al. 2010; Stephens et al. 2010; Borowitzka and Moheimani 2013; Fon Sing et al. 2013). The resulting pressures on land use make areal production rates a good gauge of the commercial viability of scale-up along with potential environmental impacts (Scott et al. 2010). Results are thus presented primarily in terms of areal production of biomass or energy-rich components (AP, AXP; gC m^−2^ t^−1^) with volumetric production (VP, VXP; gC m^−3^ t^−1^) presented in the “Supporting supplemental material”. Mean production is calculated via a trapezium rule integration of the production rate over a whole year.

## Results

### Optimizing for biomass production

**Fig. 1:**
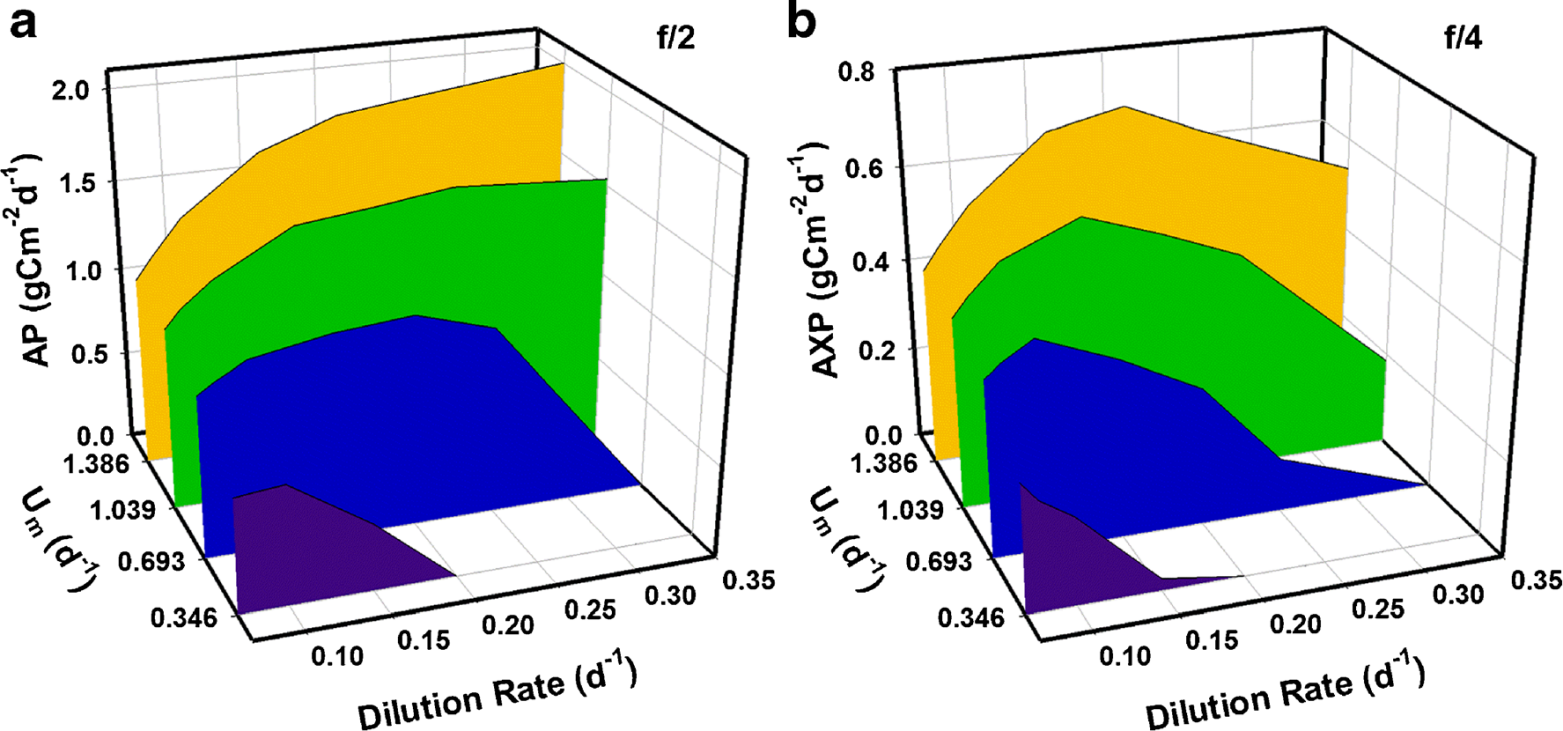
Comparison of mean daily areal production over one year from algae of the indicated maximum growth rate (*U*_*m*_), grown at different dilution rates for biomass (AP; **a**) using f/2 nutrient (see ‘Methods’ section), and excess C (AXP; **b**) using an f/4 medium to enable nutrient depletion. The optical depth is *τ* = 0.1 m and the latitude is 45°

Initial optimizations to establish appropriate dilution rates were conducted for latitude 45° with an optical depth *τ* = 0.1 m. This depth has been shown before (Flynn et al. 2010a) to provide a good compromise for balancing biomass areal and volumetric production rates (AP and VP, respectively). While absolute production rates differ for other optical depths, the overall conclusion from Fig. 1 is robust, namely that for each maximum growth rate, *U*_*m*_ (i.e. for each ‘strain’) a unique dilution rate optimises biomass yield. Figure 1a suggests that simulations of strains with *U*_*m*_ = 0.5×, 1.0×, 1.5×, and 2.0× 0.693 day^−1^ require dilution rates (*D*) of *D* = 0.1, 0.2, 0.25 and 0.35 day^−1^, respectively, in order to optimise AP. Repeating the process for excess C production (AXP; e.g. for biofuels), using a lower nutrient concentration (f/4) to promote N exhaustion, Fig. 1b shows the optimal rates are (in order of ascending *U*_*m*_) *D* = 0.07, 0.1, 0.15 and 0.2 day^−1^. As a general conclusion, optimum dilution rate under these conditions of light and nutrients for AP are of ~25 % *U*_*m*_, and for AXP of ~15 % U_m_.

**Fig. 2:**
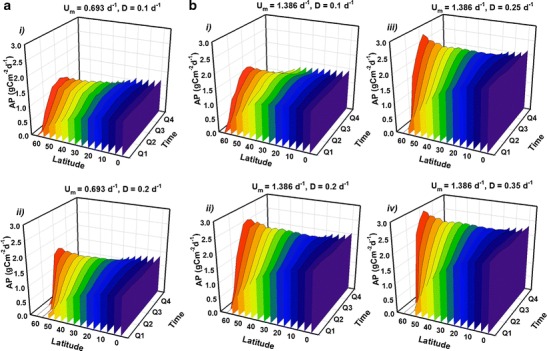
Simulated temporal and geographical variation in aereal C-biomass production (AP) over a year for strains with various combinations of maximum growth rate (*U*_*m*_) and dilution rate (*D*), as indicated with *U*_*m*_ = 0.693 day^−1^ (**a**, ‘strain S’) and 1.386 day^−1^ (**b** ‘strain F’). The year is split into four quarters, Q1–Q4, with Q1 corresponding to January, February and March in the northern hemisphere and July, August and September in the southern hemisphere, etc. Production follows seasonal changes in irradiance at each latitude. For each simulation, the optical depth is *τ* = 0.1 m and nutrients are supplied at f/2 concentrations (see ‘Methods’ section). Volumetric production rates may be inferred by multiplying AP by a factor of 1/*τ* = 10. Note the direction of the latitude scale, used to aid clarity

Guided by the initial scoping simulations (Fig. 1), further simulations were run to explore geographical and seasonal variations in AP over the course of a year for two contrasting strains cultivated at various dilution rates; a slower growing strain, ‘Strain S’ (with *U*_*m*_ = 0.693 day^−1^), and a faster growing one, ‘Strain F’ (*U*_*m*_ = 1.386 day^−1^). Nutrients were provided at f/2 concentrations and the optical depth was again fixed at *τ* = 0.1 m (hence, the equivalent results for VP in gC m^−3^ day^−1^ may be inferred by multiplying the results for AP by a factor of 1/*τ* = 10.). Results (Fig. 2a) show that, for strain S, AP at the equator remains essentially constant over the entire year, averaging around 1.2 gC m^−2^ day^−1^. Moving to higher latitudes, winter production becomes decreasingly viable. However, offsetting those losses to a degree, the summer production peak at higher latitudes is over 1.4 gC m^−2^ day^−1^ (Fig. 2a).

Seasonal and geographical variations in AP are more evident for strain F (Fig. 2b). Under optimal dilution (*D* = 0.35 day^−1^; Fig. 2b *iv*) equatorial AP production averages ~2.3 gC m^−2^ day^−1^. For latitudes 60° and above, the very small (or even zero) minimum in AP during midwinter suggests that rates much above 0.1 day^−1^ are too high, with both nutrients and cells flushed out of the system prematurely. For strain F, the peak of daily production in summer at higher latitudes may attain ca. 3 gC m^−2^ day^−1^.

**Fig. 3:**
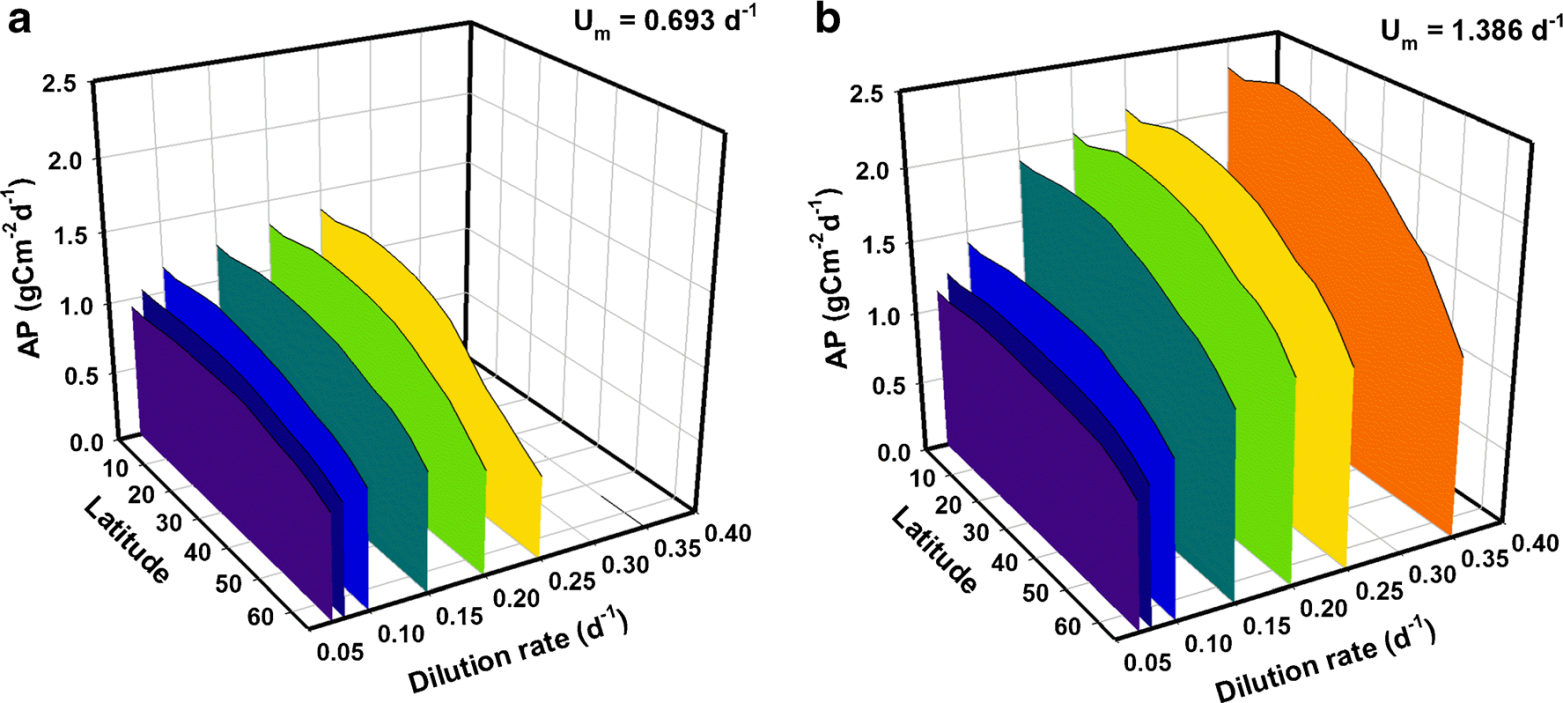
Mean daily areal productivity (AP) over 1 year for strains with *U*_*m*_ = 0.693 day^−1^ (**a**, ‘strain S’) and 1.386 day^−1^ (**b** ‘strain F’) under the full set of dilution rates from Fig. 1a. Optical depth is *τ* = 0.1 m (hence, volumetric productivity may be inferred by multiplying AP by a factor of 1/*τ* = 10) and nutrients are supplied at f/2 concentrations. Note the direction of the latitude scale, used to aid clarity

Extending the results in Fig. 2 to cover the full set of dilution rates considered in Fig. 1, Fig. 3 shows geographical variations in year-averaged AP and reveals the importance in subtle changes in the optimal dilution. For strain S (Fig. 3a), at tropical and intermediate latitudes, the mean AP ranges from 1.0 to 1.4 gC m^−2^ day^−1^ with optimal *D* = 0.2 day^−1^ whereas at high latitudes, mean production peaks at around 0.8 gC m^−2^ day^−1^ with *D* = 0.15 day^−1^. At all latitudes, strain S cells are washed out at *D* ≥ 0.35 day^−1^ and production drops to zero; this is because strain S has *U*_*m*_ = 0.693 day^−1^, which in a typical 12:12 h light/dark cycle attains a realised daily growth rate of ca. 0.693/2 day^−1^. For strain F (Fig. 3b), at tropical and intermediate latitudes, AP ranges from 1.1 to 2.3 gC m^−2^ day^−1^ under various dilution rates. At the highest latitudes, AP peaks at around 1.4 gC m^−2^ day^−1^ with *D* = 0.2 day^−1^. Under optimal conditions, AP at the highest latitude tested (65°) in both cases is approximately 60 % of rates predicted for equatorial latitudes.

**Fig. 4:**
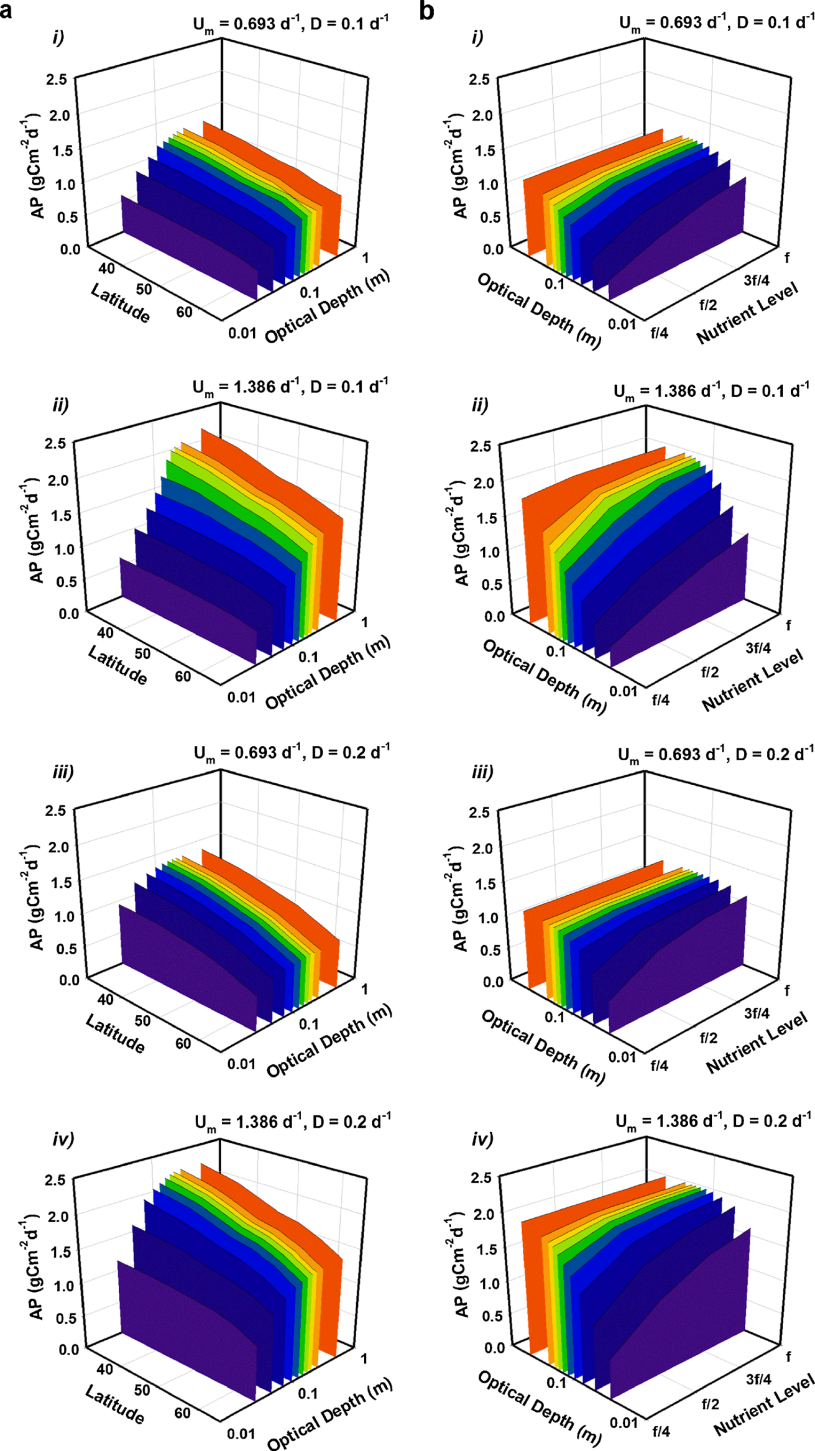
Predicted mean daily C-biomass areal productivity (AP) over 1 year versus optical depth with latitude (**a**) or with nutrient availability (**b**), Simulations are shown for strains with *U*_*m*_ = 0.693 day^−1^ (‘strain S’, panels *i* and *iii*) and 1.386 day^−1^ (‘strain F’, *ii* and *iv*) under dilution regimes *D* = 0.1 day^−1^ (panels *i* and *ii*) and *D* = 0.2 day^−1^ (*iii* and *iv*). In **a**, the latitude ranges from 30° to 65° with supply of f/2 nutrient (see ‘Methods’ section). In **b**, the latitude is 45° with nutrient supplied as indicated. Optical depth, shown on a log scale, ranges from 0.03–0.5 m. AP saturates as optical depth and nutrient availability are increased. (The corresponding results for volumetric production are presented in Fig. S1 in the supporting material)

Light availability is a major factor limiting algal growth and varies either through solar elevation or absorption by the microalgal suspension. Figure 4a shows how, over non-tropical latitudes (30° to 65°; a range covering most of Europe, North America and Asia in the Northern hemisphere and Southern Australia, New Zealand, Chile and Argentina in the Southern hemisphere), mean AP saturates at *τ* ~0.25 m, constraining the maximum useful pond depth for AP (see Fig. S1a in the Appendix for the contrasting effects on VP). Other saturation effects are seen in Fig. 4b, for latitude 45°, as depth and nutrient concentration are altered (contrasting with the results for VP; see Fig. S1b in the supporting material). The culture transitions from nutrient-limited conditions below f/2 concentrations to light-limited conditions for τ > 0.2 m. (The relationship between nutrient concentration, optical depth, dilution rate and the transition between light and nutrient limitations are further portrayed in Fig. S2 in the electronic supporting material).

### Production of energy-rich components

**Fig. 5:**
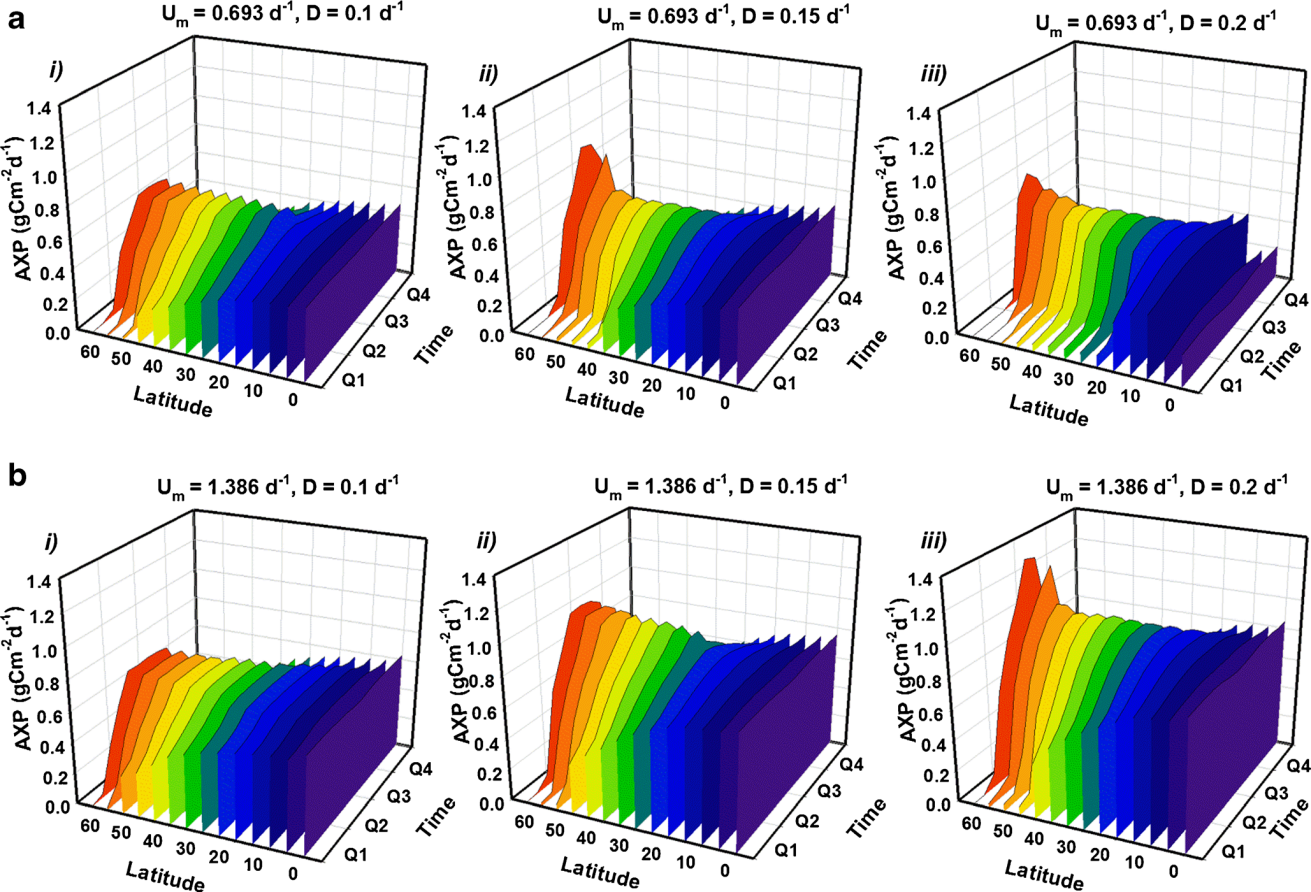
As in Fig. 2 but for production of excess C (AXP) with *U*_*m*_ = 0.693 day^−1^ (**a** ‘strain S’) and 1.386 day^−1^ (**b** ‘strain F’). Here, while the optical depth is still *τ* = 0.1 m, the nutrients are supplied at half the concentration as for biomass (Fig. 2), using an f/4 medium. (Volumetric production may be calculated by multiplying AXP by 1/*τ* = 10). Note the direction of the latitude scale, used to aid clarity

Optimizing production of Cex_C_ differs from that of biomass in that it is imperative that nutrients are exhausted, and that light limitation does not become significant. Guided by the initial results for optimisation of AXP (Fig. 1b), equivalent plots to those for biomass (shown in Fig. 2) were generated by simulating Cex_C_ production over a year at various latitudes (Fig. 5). Here, the optical depth is again set at *τ* = 0.1 m, but in order to ensure nutrient exhaustion, the nutrient concentration is halved from f/2 to f/4. Results reveal seasonal and geographical variation in daily AXP (again, the corresponding VXP in Cex_C_ m^−3^ day^−1^ may be calculated by multiplying AXP by a factor of 1/*τ* = 10 while the associated areal biomass production, AP, at f/4 is shown in Fig. S3 in the Appendix). For strain S, AXP at tropical latitudes averages around 0.5 g Cex_C_ m^−2^ day^−1^ while strain F yields up to 0.8 g Cex_C_ m^−2^ day^−1^. Setting the dilution rate at 0.15 day^−1^ induces a sharp peak in AXP for strain S in mid-summer at latitudes >60°, attaining 1 g Cex_C_ m^−2^ day^−1^. For strain S at *D* = 0.2 day^−1^ (Fig. 5a *iii*) there appears to be a suppression of AXP at very low latitudes. An analysis of the quotient describing N sufficiency (see (Flynn 2002) for further details) revealed that, at latitudes 0–5°, the system is running in a state very close to the interface between light and N limiting conditions. This illustrates how small changes in culture conditions can have large effects on AXP. The sharp midsummer peak in AXP at high latitudes is repeated for Strain F at this dilution rate (*D* = 0.2 day^−1^; see Fig. 5b *iii*). The long midsummer days decrease the potential for day-integrated light limitation (noting that simulations included light–dark periodicity and not just changes in the day-integrated irradiance dose); there is a projected window of opportunity for a few months to maximise daily yields with a peak of 1.3 g Cex_C_ m^−2^ day^−1^.

**Fig. 6:**
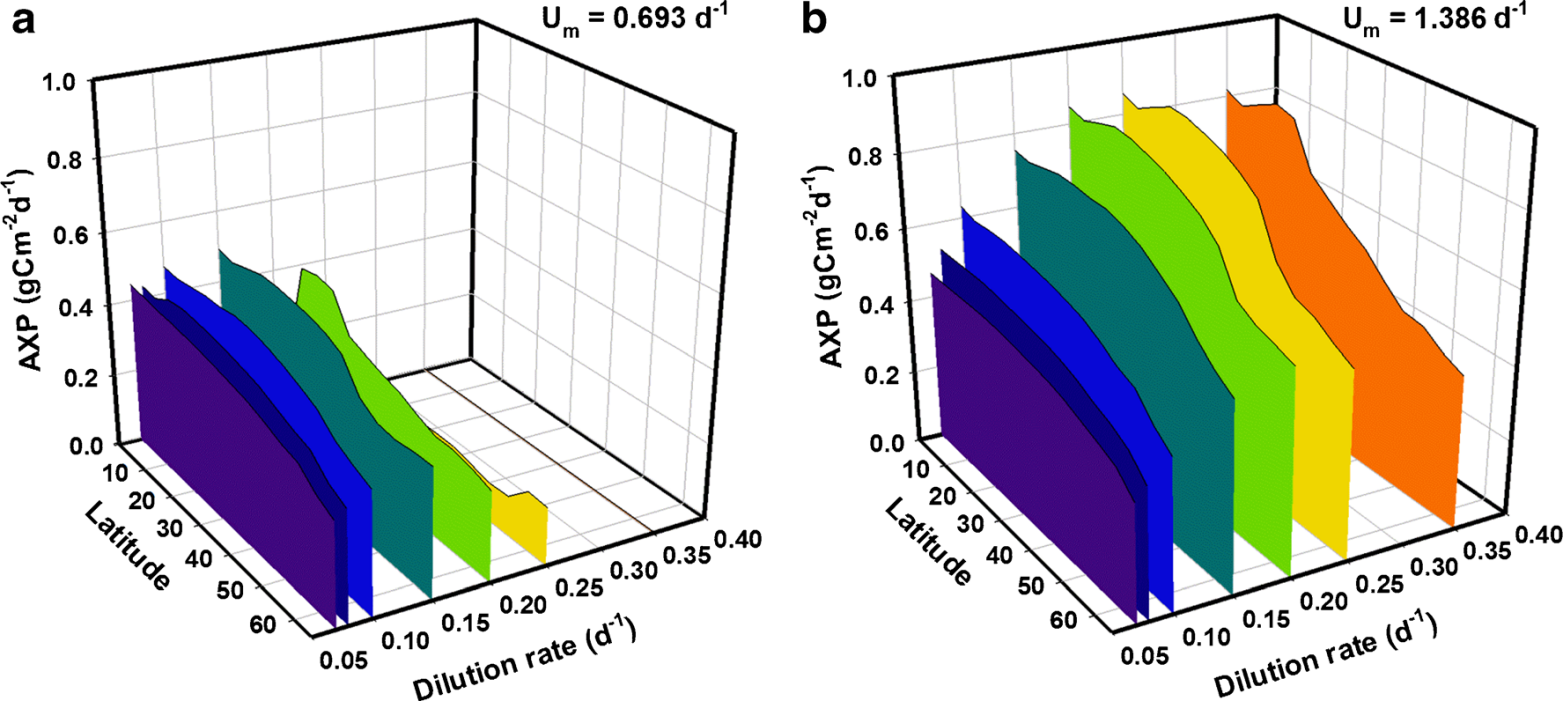
As in Fig. 3 but for mean daily production of excess C (AXP) over 1 year (**a** ‘strain S’, **b** ‘strain F’). Here, while the optical depth is still *τ* = 0.1 m, the nutrients are supplied at half the concentration as for biomass (Fig. 3), using an f/4 medium. (Volumetric production may be calculated by multiplying AXP by 1/*τ* = 10). The resulting areal biomass production (AP) under these conditions is presented in Fig. S3 of the supporting material and a comparison of AP and AXP implies a peak percentage Cex_C_ content of 63 % for *D* ≤ 0.08 day^−1^ at low latitudes. Note the direction of the latitude scale, used to aid clarity

Mean AXP of strains S and F for the full set of dilution rates over latitudes 0° to 65° is plotted in Fig. 6. For strain S, AXP is highly insensitive to changes in dilution rate in the range considered up to *D* = 0.15 day^−1^, where it peaks around 0.5 g Cex_C_ m^−2^ day^−1^ at tropical and intermediate latitudes, and nearer 0.35 g Cex_C_ m^−2^ day^−1^ over higher latitudes. Beyond this dilution rate, production drops away until washout occurs as *D* approaches 0.35 day^−1^. In contrast, for strain F, a dilution rate of around 0.2 day^−1^ appears optimal for high AXP, providing a peak in excess of 0.8 g Cex_C_ m^−2^ day^−1^ at tropical and intermediate latitudes, falling towards 0.55 g Cex_C_ m^−2^ day^−1^ at high latitudes. Once more, mean AXP at the highest latitudes is considerably less than that nearer the equator, achieving between 60 and 70 % of the values predicted at the lowest latitudes.

**Fig. 7:**
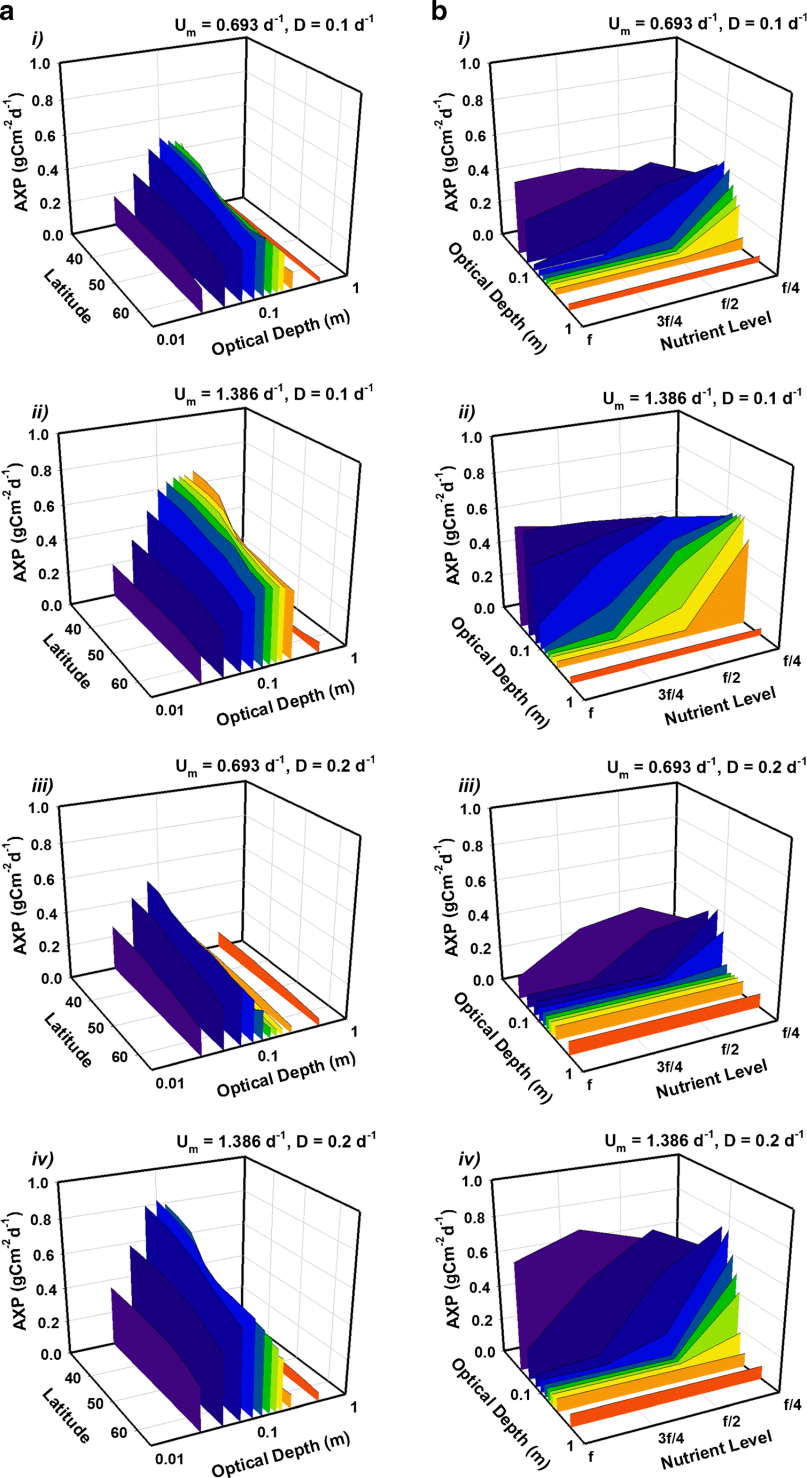
As in Fig. 4 but for production of excess C (AXP). In contrast with biomass production (CF Fig. 4a), **a** shows when nutrient concentrations are halved (f/4 medium) AXP peaks at a critical optical depth *τ* ~0.1 m. Another contrast is seen in **b** (CF Fig. 4b), at 45°, with AXP falling rapidly as depth and nutrient availability are increased. Optical depth, shown on a log scale, ranges from 0.03–0.5 m. (Volumetric production rates are presented in Figs. S4 and S5 in the supporting information)

An interesting contrast to the biomass analysis can be seen in Fig. 7a, where mean AXP is plotted for a set of latitudes covering most of the temperate zone (30° to 65°), examining its dependency on the optical depth. For biomass, self-shading led to saturation effects on AP with increasing optical depth (see Fig. 4). However, while AXP increases proportionally with optical depth for shallow depths, there appears to be a well-defined (in most cases) critical depth at ~0.1 m at which production peaks. The effect is pronounced for all latitudes considered, suggesting that the selection of a suitable optical depth is at least as important as location for production of microalgae with high lipid/carbohydrate (e.g. biofuels) content.

A comparison, at 45°, of AXP (Fig. 7b) with AP (Fig. 4b) emphasises the importance of establishing the optimum optical depth. Projected AXP is plotted against optical depth and nutrient availability, illustrating well the opposing conditions that are effectual for AXP vs AP. Note that the direction of the *x* and *y* axes in Fig. 7b are (for clarity) reversed compared to those in Fig. 4b. Differences reflect the need for nutrient exhaustion for high rates of biofuel production, versus the need for high nutrient levels (which then result in light limitation through self-shading) required for enhanced biomass production.

**Fig. 8:**
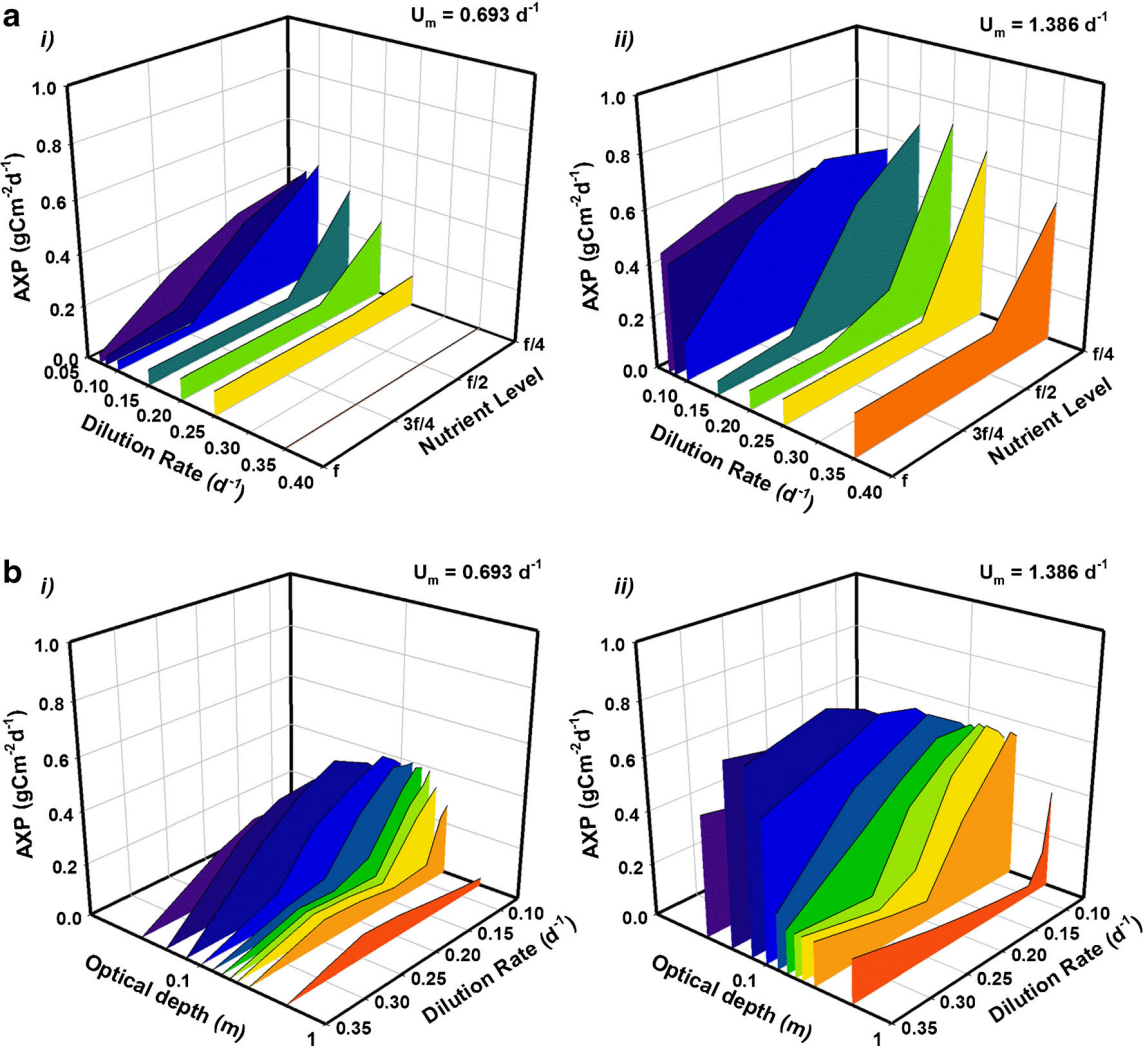
Mean daily areal production of excess C (AXP) over 1 year at latitude 45° for strains with maximum growth rates (*U*_*m*_) of 0.693 day^−1^ (‘strain S’; **a** and **b** *i*) and 1.386 day^−1^ (‘strain F’; **a** and **b** *ii*) versus nutrient availability and dilution rate (**a**) with optical depth *τ* = 0.1 m and versus dilution rate and optical depth (**b**) and with an f/4 nutrient medium. Optical depth in **b**, shown on a log scale, ranges from 0.03–0.5 m. (Volumetric production rates for **a** may be inferred by multiplying by a factor of 1/*τ* = 10 and for **b** are presented in Fig. S6 in the supporting information.)

To further explore this conflict, when AXP is plotted against nutrient availability and dilution rate (Fig. 8a), it becomes apparent that a slower dilution rate plus nutrient deficiency stimulates accumulation of C-rich components. Beyond this zone of low nutrient/slow dilution, any increase in AXP is just a consequence of accumulating extra biomass, but the increase in AXP is much less than the corresponding increase in AP. Hence, while the use of strains with a high *U*_*m*_ remains the most important single base factor, AXP can be further enhanced through a judicious choice of dilution rate and nutrient concentration.

Figure 8b compares simulated annual AXP of strains S and F under nutrient-deficient conditions (using an f/4 medium) at a virtual facility located at a latitude of 45°. The dilution rate is varied for a number of systems with depth ranging from 0.05 to 0.5 m. For strain S, a dilution rate of around 0.1 day^−1^ combined with a shallow depth of 0.1 m (favouring nutrient rather than light limitation) provides a peak in mean AXP of over 0.4 g Cex_C_ m^−2^ day^−1^. Cultivating strain F appears to give more scope for operational flexibility with enhanced AXP possible in systems with *τ* ≤ 0.2 m if the dilution rate is restricted to a maximum of 0.1 day^−1^, whereas the dilution rate can be as high as 0.25 day^−1^ for *τ* < 0.1 m before areal production falls away. Peak annual AXP of a fast-growing stain in this virtual system is approximately 0.8 g Cex_C_ m^−2^ day^−1^ and occurs in a 0.075 m deep pond with *D* = 0.25 day^−1^.

## Discussion

### Comparing the model projections with empirical evidence

This work seeks to provide a more quantitative assessment of the scope for solar-powered algal biomass production than has hitherto been published. Absolute peak of year-averaged C-biomass productivity in these simulations was 2.4 gC m^−2^ day^−1^. More typically, mean AP (Fig. 3) fell between 0.8 and 2.3 gC m^−2^ day^−1^, depending on strain configuration and geographic location. Taking the C/dry weight biomass ratio to be 31 % (Heymans 2001) and assuming uninterrupted production could be maintained, this allows a rough estimate of a limit on annual areal production at slightly under 30 t dw ha^−1^ year^−1^ for a strain which undergoes one doubling during a 12:12 h light/dark cycle. For strains with faster maximum growth rates (along with a suitable choice of dilution rate), the absolute value of production should rise further.

This projected peak value of 30 t dw ha^−1^ year^−1^ from our simulations falls within the mid-range of those reported for different real systems. It is in good agreement with the results of Jimanez et al. (2003) who report annual production of 30 t dw ha^−1^ year^−1^ in raceways in Southern Spain. Olguin et al. (2003) report an average production of 11.8 g m^−2^ day^−1^ in Mexico over the course of a year cultivating *Spirulina* using animal waste, which would equate to around 40 t dw ha^−1^ year^−1^ of biomass if production could remain uninterrupted over the whole year. Productivity of 60 t dw ha^−1^ year^−1^ of *Pleurochrysis carterae* in flat ponds has been reported (Moheimani and Borowitzka 2006), and cited as an example of maximal productivity (Williams and Laurens 2010), but this weight includes 10 % of calcium carbonate in the form of coccoliths and remains very much the exception rather than the rule. At the other end of the scale, García-González et al. (2003) achieved production of *Dunaliella* equating to around 6 t dw ha^−1^ year^−1^. This compares favourably with the projected AP for the slowest growth rate optimised for in Fig. 1 (0.65 gC m^−2^ day^−1^ for *U*_*m*_ = 0.346, comparable to that of *Dunaliella*) which is approximately 7.5 t dw ha^−1^ year^−1^. For other system types, there are reports of AP up to 60 t dw ha^−1^ year^−1^ for tubular PBRs (Fernández et al. 1998; de Schamphelaire and Verstraete 2009; Rodolfi et al. 2009) and nearly 40 t dw ha^−1^ year^−1^ for hybrid systems (Huntley and Redalje 2007), dependent upon the strain cultivated. The more typical model predictions for AP above of 3–8.4 tC ha^−1^ year^−1^ (around 10–27 t dw ha^−1^ year^−1^ using the above estimation) are of the same order as estimated by Ritchie and Larkum (2012) who measure net photosynthesis for three algae species from measurements of light attenuation in cultures of varying optical depths. In conclusion, this all instills confidence that the model used in this study is producing plausible projections.

### Potential for biofuels production

Our simulations indicate a maximum potential biofuels production rate of 0.9 g Cex_C_ m^−2^ day^−1^, attainable at latitude 15° with a system of optical depth of 0.1 m and a dilution rate of *D* = 0.25 day^−1^, under an f/4 nutrient regime (see Fig. 6b). This assumes that all Cex_C_ is of use for biofuels production. The maximum Cex_C_ content of the simulated microalgae was 63 % of algal C-biomass (compare Fig. 6 with Fig. S3) but this coincided neither with peak AP (with 9 % Cex_C_) or peak AXP (48 % Cex_C_). The typical Cex_C_ content using f/4 medium ranged between 10 and 60 % for strain S and 30 and 60 % for strain F, depending on latitude and dilution rate. For comparison with strain S, lipid content of up to 60 % has been measured in strains of *Nannochloropsis* under N deprivation (Rodolfi et al. 2009) whereas for *Scenedesmus* (cf. strain F) optimised lipid content has been reported at 58 % (Mandal and Mallick 2009). A review of reported lipid content values is provided by Mata et al. (2010).

The shallow nature of the optimal depth required to assure a production of biofuels becomes a challenge if considering flat raceways for cultivation; requirements for adequate mixing and high susceptibility to evaporation and temperature fluctuations in such shallow ponds mean that it is often impractical to operate raceway systems with depths less than 0.15 m (Tredici 2007; Ritchie and Larkum 2012). Whilst not detrimental to AP per se, the subsequent lower VP resulting from this pragmatic limitation decreases the potential profitability by increasing demand for water and nutrients and increasing harvesting costs. Furthermore, the results in Fig. 7a imply there is also a direct adverse effect on AXP. Increasing the optical depth to 0.15 m (while keeping *D* fixed) leads to a decrease in AXP of between 10 and 25 % (depending on latitude) compared to the potential peak value. Increasing depth further to 0.2 m results in a halving (or worse) of AXP compared to production under optimal conditions. To some extent, this can be mitigated by adjusting the dilution rate appropriately, as Fig. 8b shows; slowing the dilution rate from *D* = 0.25 to 0.1 day^−1^ limits the decrease in AXP from peak values to about 20 %. Even so, if the system which produced the peak value in AXP quoted above was limited in practice to a depth of 0.2 m with dilution slowed to *D* = 0.15 day^−1^, peak AXP would not exceed 0.8 g Cex_C_ m^−2^ day^−1^.

These factors, and given that the model is producing results consistent with data from real systems (“Comparing the model projections with empirical evidence” section), appears to provide a robust estimation of the upper potential for solar-powered microalgal biofuels production of 3 t biofuels ha^−1^ year^−1^, which equates to ~4,000 L ha^−1^ year^−1^ assuming a carbon fraction of 720 gC L^−1^ (which is typical for diesel fuels (Miguel et al. 1998)) and that all of the excess C can be recovered and is in the form of lipids. While outperforming many land-based crops, these results imply algae are not appreciably more productive for biofuels and can be even less so in comparison with, as an example, palm oil (Chisti 2007; Schenk et al. 2008; Mata et al. 2010; Scott et al. 2010). This upper limit is in agreement with the calculation performed by Walker (2009) and far below many estimates of theoretical limits (Weyer et al. 2010). In reality, it is unlikely (if not impossible) that optimal culturing conditions can be maintained long enough to achieve the kind of results for biomass and biofuels production obtained in these simulations. To be able to quantify this further requires a detailed sensitivity analysis of risk factors. Even so, should it be possible to overcome the technical difficulties, the physiological limits on cell growth constrain the potential for algae as a feedstock for biofuels.

As a result, the potential for biofuels production from microalgae appears of questionable commercial viability, unless a step change can be attained in algal physiology through GM, with all of its attendant risks. For instance, Flynn et al. (2013) demonstrated through simulation how engineering strain characteristics to allow greater capacity for photosynthetic efficiency coupled with a decrease in the maximum Chl:C ratio could boost productivity by up to five times that of natural strains. They projected a maximum Cex_C_ production rate of AXP = 7.5 g Cex_C_ m^−2^ day^−1^ = 20,000 L ha^−1^ year^−1^ of biodiesel. At the same time, they also demonstrated how the creation of such unpalatable, highly productive strains (desirable traits for biofuels production) could easily lead to harmful, even catastrophic, blooms if they escaped into nature.

To date, even with more optimistic production estimates, there remains much uncertainty for the economic potential for microalgal biofuels production (Liu et al. 2012; Sills et al. 2013). In life cycle analyses, this uncertainty is dominated by sensitivity to the algae’s lipid content and growth rate (Stephenson et al. 2010; Davis et al. 2011). Unfortunately, the biological modelling components within otherwise complicated LCA scenarios are invariably based on assumptions and generalisations derived from literature which lead to projections of several hundreds of tons of biomass produced per hectare per year (Williams and Laurens 2010) whereas in practice (as seen from the references above) 60 t dw ha^−1^ year^−1^ is the highest claimed to date. Even if 60 t could become the rule rather than the exception, only a fraction of that (ca. at most 50 %) can be expected to constitute stock for biofuels. Our results indicate production of biomass AP below 30 t dw ha^−1^ year^−1^ and biofuels feedstock AXP up to 8 t dw ha^−1^ year^−1^.

In consequence of all of these interacting events, conducting a full LCA on the commercial viability of the whole process (whether for biomass, biofuels or other products) requires an integrated approach taking into account the physiology of the microalgae that lay at the heart of the whole endeavour. At present, LCAs take scant regard of this issue and in consequence may be at significant variance from reality. It is likely that the inherent uncertainty will remain unresolved until LCAs become coupled to mechanistic models (such as the one used here) that can more adequately capture the dynamic physiological subtleties of microalgal growth. Combining these informative but differing computational approaches can provide a powerful tool that will allow operators to explore realistic options leading towards improved production.

### Areal vs volumetric production

The emphasis above has been upon areal production of biomass (AP) and of excess C as biofuels (AXP). In the Appendix are the corresponding volumetric production values (VP, VXP, respectively). For commercial operations, it is important to maintain an optimum balance between AP and VP. However, this ideal is conflicting as the highest VP requires very low optical depths, which do not then permit high AP (see Flynn et al. 2010a). In oceans, with optical depths of many tens of meters, VP is extremely low, but AP by fast-growing phytoplankton at upwelling zones (ca. 3–4 gC m^−2^ day^−1^ (Field et al. 1998)) can match rates in shallow ponds (Flynn et al. 2013) in short bursts during spring blooms. Figure S1 illustrates how a high VP requires a shallow optical depth to prevent light limitation and this has the added benefit of diminishing the demands for nutrients and water, and hence harvesting costs. The trade-off comes as the resulting low volume minimises AP. Increasing depth to boost AP suppresses VP but the rate of depth increase initially outpaces the rate of VP decrease and so the AP continues to rise. After a certain point, VP decreases in proportion to the increase in depth which leads to the saturation of AP seen in Fig. 4.

For Cex_C_, the corresponding rise in AXP (Fig. 7) as VXP falls (Fig. S4a) initially follows the trend for AP as optical depth increases. However, beyond a critical optical depth (~0.1 m) light rather than nutrient becomes the limiting factor; Cex_C_ production is suppressed and VXP decreases faster than the depth increases leading to the fall in AXP. Production of C-rich products (e.g. biofuels) is, therefore, more sensitive to the conflict between areal and volumetric production than is biomass production. This conflict will extend directly to costs for space (and/or PBR infrastructure) and in preparing and handling different volumes of water/algal suspension and nutrient loadings. Flynn et al. (2010a) described this interaction using a function relating AP and VP to what they termed a commercial production index. While this provided a single index, in the commercial world the costs of land, energy, nutrients, and other resources would apply differential weights to AP vs VP.

The results from the simulations presented here enable a number of useful conclusions to be drawn in this regard. Most notably, the routes to maximising production of biomass are not the same as those for maximising fatty acid/biofuels production. That said, while individual needs may vary, the optimal depth for commercial cultivation of wild-strain (non-GM) phototrophic microalgae in a facility intended for multiple applications should be approximately 0.1 m (a value consistent with that suggested by García-González et al. (2003) and Ritchie and Larkum (2012) who also place an upper limit on useable pond depth around 25 cm) coupled with the use of nutrient loads of around f/2 containing 12.35 mg N L^−1^ and 1.11 mg P L^−1^ (Guillard and Ryther 1962) for biomass production, and f/4 for biofuels production. Further, in general, stimulating biofuels production requires the combination of a fast-growing strain, and nutrient deficiency, which is promoted by shallow optical depth and relatively slow dilution rates.

### Latitudinal impacts

Not surprisingly, production at high latitudes is projected to be far more seasonally dependent than at lower latitudes, as seen for both biomass in Fig. 2 and Cex_C_ in Fig. 5. However, while biomass production in mid-winter may be so poor as to likely not be commercially viable, the longer summer days have potential to provide a window for increased production over the summer months sufficient to ensure viability. That may be especially so if the intended use of the biomass in support of seasonal aquaculture activities. This paints a qualitatively similar picture to the gross photosynthesis calculations of Ritchie (2010) and to Williams and Laurens (2010) who suggest a lack of sufficient irradiance over winter months restricts areal production at high latitudes to little more than half of that possible at equatorial latitudes, compared to our prediction of around 60 % of maximum. However, Williams and Laurens’ estimate of absolute values for production (obtained by assuming either a 3 or 10 % bioenergetic yield with a biomass calorific value of 24.7 kJ g^−1^ dw) are larger than the mechanistic model calculations by a factor of 10. Our values are more keeping with empirical values from the literature (‘Comparing the model projections with empirical evidence’ section).

The optimal dilution rate depends primarily on the maximum growth achievable by the strain cultivated. Even though growth may be conducted at low dilution rates, a high *U*_*m*_ is a desirable trait to select or engineer into microalgal strains (Flynn et al. 2013). However, this trait is likely to be selected against during long-term enforced slow growth in continuous culture systems (Flynn 2009). Away from tropical latitudes, location becomes an additional factor. The extent to which it does so also depends upon the maximum growth rate; production using slower growing strains is more sensitive to the choice of dilution rate with increasing latitude than it does for a faster growing strain; the optimal dilution rate progressively decreases the further from the equator the facility is situated (Figs. 3 and 6). Furthermore, as faster-growing strains are less sensitive to the choice of dilution rate, the most appropriate dilution rate may not necessarily be the one that supports maximum biomass yield. Figure 1a shows that decreasing the dilution rate for the strain with *U*_*m*_ = 1.386 day^−1^ from *D* = 0.35 to 0.2 day^−1^ uses <60 % of the nutrients and water but still returns 90 % of peak AP (1.8 cf. 2 gC m^−2^ day^−1^). Such a saving of resources is likely to impact significantly on commercial viability, especially as nutrient prices increase.

### System optimisation

The results from Fig. 1, and indeed the seasonal variability in production at high latitudes, imply a single objective optimisation method may be insufficient. A more sophisticated and effective method could be to use multi-objective optimisation to balance between maximising biomass production and minimising resource consumption, and also consider financial inputs and outputs. At extremes, one could consider changing algal strains or system optical depths (Olguin et al. 2003; Moheimani and Borowitzka 2007), but more readily changed are facility operational procedures such as dilution rate.

While higher-plant crops are grown in what amounts to discontinuous culture, industrial-scale microbial growth is most commonly within continuous culture systems. All of the simulations performed here have been run in such a chemostat mode, with continuous dilution and harvesting at a constant rate. While there are problems with using such an approach (notably strain selection to match maximum growth rate to dilution rate, and the risk of establishing pest-predators), there are also distinct logistic problems in discontinuous approaches. These include the necessity to rapidly drain and harvest large volumes of medium containing the biomass, and replace the same volume with fresh medium and nutrients. Exposure of the newly diluted remaining culture to high light then risks photodamage, especially if the organisms are nutrient stressed (Geider et al. 1993).

Improvements to the use of continuous dilution methods as simulated here would be to consider seasonal changes to the dilution rate and/or through the introduction of discontinuous harvesting methods into the simulations. From our results, it appears that an optimisation of dilution rates separately for winter and summer months could be sufficient. For instance, from the results in Fig. 2b *i* and *iv*, it seems more appropriate to run with a substantially slower dilution rate in winter at high latitudes and increase dilution as production ramps up later in the year.

To fully automate the optimisation process, a real-time regulation of production rates is needed. Computationally, this can be achieved using a simple predictor-corrector. At regular intervals, a prediction is made of the output at the end of a time period *t* + m∆*t* based on the current dilution rate and compared to the output for a set of dummy rates. Whichever gives the best result becomes the rate for that time period. Methods of intelligent harvesting (whether manual or automated) would have to rely on real-time monitoring of culture conditions, with several measurements taken simultaneously. Monitoring and regulation of external nutrient levels would be quite straightforward, deploying nutrient probes directly exposed to the culture medium. Regulating dilution based on algal physiology is more problematic, though monitoring of biomass and photophysiology using *F*_*v*_/*F*_*m*_ to monitor the efficiency of the PSII photosystem are obvious starting points. Measurement of the fluorescence emission spectrum can reveal levels of nutrient stress in the culture (Masojídek et al. 2000); a threshold value could be used as a trigger for further nutrient injection. Light absorption is an indicator of total biomass (Griffiths et al. 2011) and measurement of changes in turbidity can provide an estimation of growth rates, while analysis of the absorption spectrum can quantify the amount of chlorophyll per unit of biomass. Thus, if a measurement of turbidity and/or *F*_*v*_/*F*_*m*_ indicates an aberrant change in production then the dilution rate can be automatically adjusted to compensate.

Intelligent control of dilution rates and nutrient addition is one way to optimise yields, but a more radical control scenario would enable switching between periods of continuous and discontinuous operation. This leads to further questions as to what the optimal dilution and harvesting strategies may involve in different modes, and how these may relate to other factors such as the control of pests. These topics will be considered in future papers.

## Electronic supplementary material

Below is the link to the electronic supplementary material.

ESM 1 (PDF 2040 kb)
