# Supplementary material for: In silico optimization for production of biomass and biofuel feedstocks from microalgae
Source: J Appl Phycol. 2014 Jun 1;27(1):33–48. doi: 10.1007/s10811-014-0342-2 (PMC4297880; doi:10.1007/s10811-014-0342-2)
Supplement: Supplementary file 1 — (PDF 2040 kb) [file 10811_2014_342_MOESM1_ESM.pdf]

Appendix: Supplementary Material

Varied Parameters			
Parameter	Description	Value Range	Unit
τ	Optical depth	0.03 - 0.5	m
D	Dilution	0.07 – 0.35	d^{-1}
U_m	Maximum growth rate	0.346 – 1.386	d^{-1}
latitude		0 - 65	degrees
Nutrient-N		(0.5 – 2.0)×12.35	gN m^{-3}
Nutrient-P		(0.5 – 2.0)×1.12	gP m^{-3}
**Fixed Parameters**			
Parameter	Description	Value	Unit
$ChlC_m$	maximum pigment content per g of cell-C	0.06	gChl $(gC)^{-1}$
α_{Chl}	initial slope of photosynthesis–irradiance curve	7×10^{-6}	(gC mol^{-1} photon) ×(m^2 g^{-1} Chl)
M	controls photoacclimation rate	2	dimensionless
NC_0	minimum N quota	0.05	gN $(gC)^{-1}$
PC_0	minimum P quota	0.005	gP $(gC)^{-1}$
KQN	controls efficiency of cell-N usage	10	dimensionless
KQP	controls efficiency of cell-P usage	0.01	dimensionless
BasRes	basal respiration as a fraction of U_m	0.05	dimensionless
ProtRes	metabolic respiration referenced to N-growth	1.5	gC respired × $(gN\ assimilated)^{-1}$
CstrucN	structural C relative to nitrogenous core	0.8	gC $(gN)^{-1}$
CNcore	C:N for the nitrogenous core of the cell (protein, nucleic acids, etc.)	3.2	gC $(gN)^{-1}$

Table S1 Model Parameters, either varied for optimisation or fixed according to literature values (see main text and Flynn et al. (2013) for further explanation).

Supplemental Figures:

The following plots are supplemental to the main article. They mainly comprise the volumetric equivalent of the areal production plots in the main text. Areal and volumetric biomass production are designated by AP and VP respectively while for excess-C ($Cex_{C,}$ eg. biofuels) production these are abbreviated to AXP and VXP

Fig. S1 Predicted mean VP over one year versus latitude (from 30° to 65°) and optical depth with fixed concenteration of f/2 nutrient (plot a) and versus nutrient availability and optical depth at latitude 45° (plot b) for simulated microalgae with maximum growth rates (U_m) of 0.693 d^{-1} and 1.386 d^{-1}, each under dilution rates D = 0.1 d^{-1} and 0.2 d^{-1}, as indicated (cf. AP in Fig. 4 in the main text).

Fig. S2 Mean AP over one year versus nutrient availability and dilution rate with a fixed optical depth of τ = 0.1 m (plot a, allowing VP to be inferred by multiplying AP by a factor of $1/\tau$ = 10) and versus dilution rate and optical depth with fixed f/2 nutrient (plot b) at latitude 45° for simulated microalgae with maximum growth rates of 0.693 d^{-1} and 1.386 d^{-1}. AP saturates with increasing optical depth. VP corresponding to plot b) is shown in plot c).

Fig. S3 Mean AP over one year versus latitude and dilution (cf. Fig. 6 in the main text) with nutrients supplied by an f/4 medium and an optical depth of τ = 0.1m (hence the corresponding VP may be calculated by multiplying by a factor of $1/\tau$ = 10). A comparison with AXP in Fig. 6 implies a peak percentage Cex_C content of 63% for D $\leq$ 0.08 d^{-1} at low latitudes.

Fig. S4 Mean VXP over one year (plot a) plus associated VP (plot b) and AP (plot c) resulting from variations in latitude and depth (cf. Fig. S1a) with dilution and U_m as indicated. Nutrients are supplied by an f/4 medium, at half concentration compared to Fig. S1. (See also Fig. 7a in the main text for AXP)

Fig. S5 As Fig. S1b but for VXP with nutrients supplied by an f/4 medium. The corresponding AXP is plotted in Fig. 7b in the main text.

Fig. S6 Mean VXP over one year (plot a) plus VP (plot b) and AP (plot c) versus dilution rate and optical depth at latitude 45° for strains with maximum growth rates of 0.693 d^{-1} and 1.386 d^{-1}. Nutrients are supplied by an f/4 medium (cf. Fig 8 in the main text).

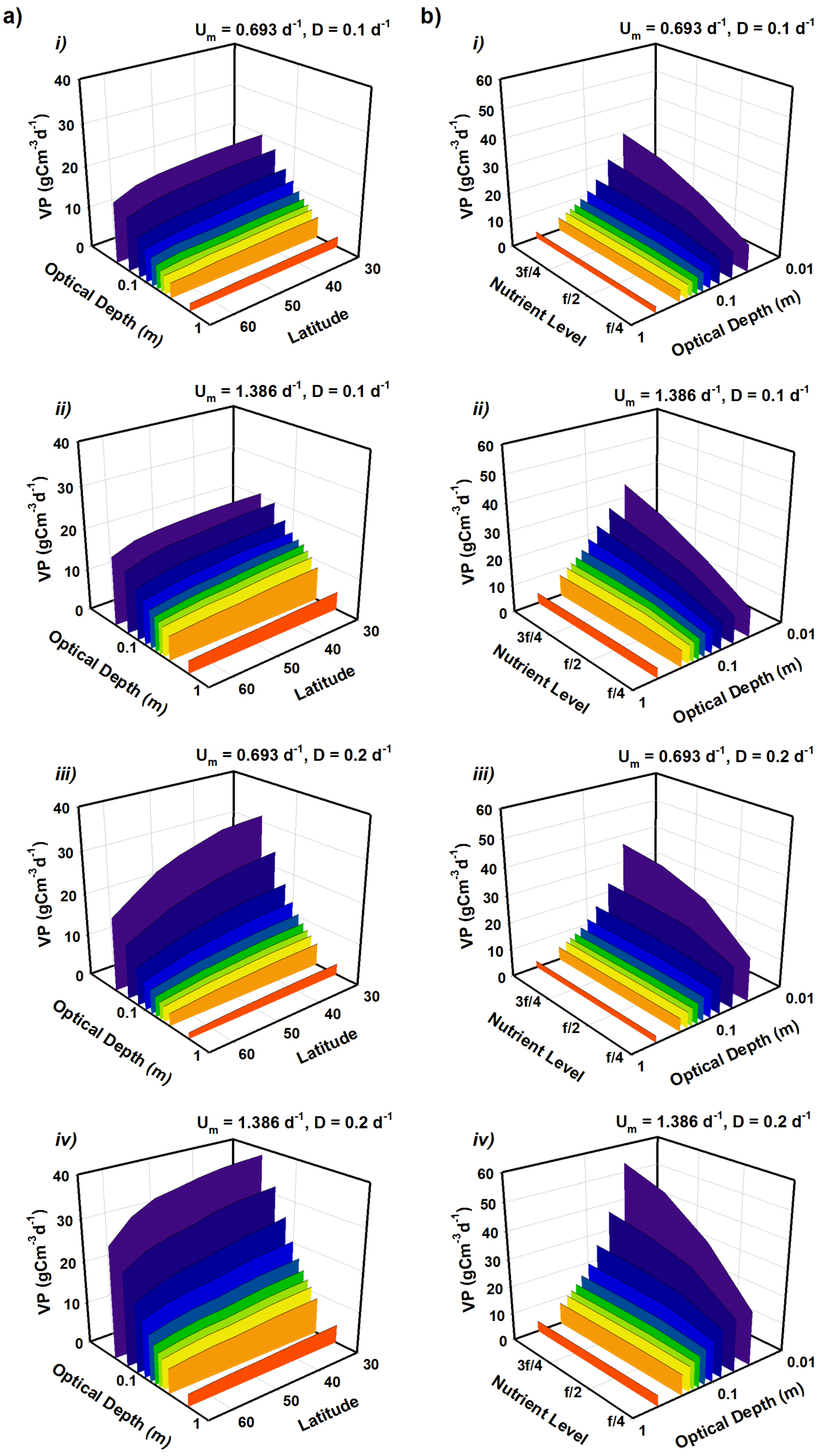

a)
b)
i)
ii)
iii)
iv)
U$_m$ = 0.693 d^{-1}, D = 0.1 d^{-1}
U$_m$ = 1.386 d^{-1}, D = 0.1 d^{-1}
U$_m$ = 0.693 d^{-1}, D = 0.2 d^{-1}
U$_m$ = 1.386 d^{-1}, D = 0.2 d^{-1}
VP (gCm^{-3}d^{-1})
Optical Depth (m)
Latitude
Nutrient Level
3f/4
f/2
f/4

Figure S1

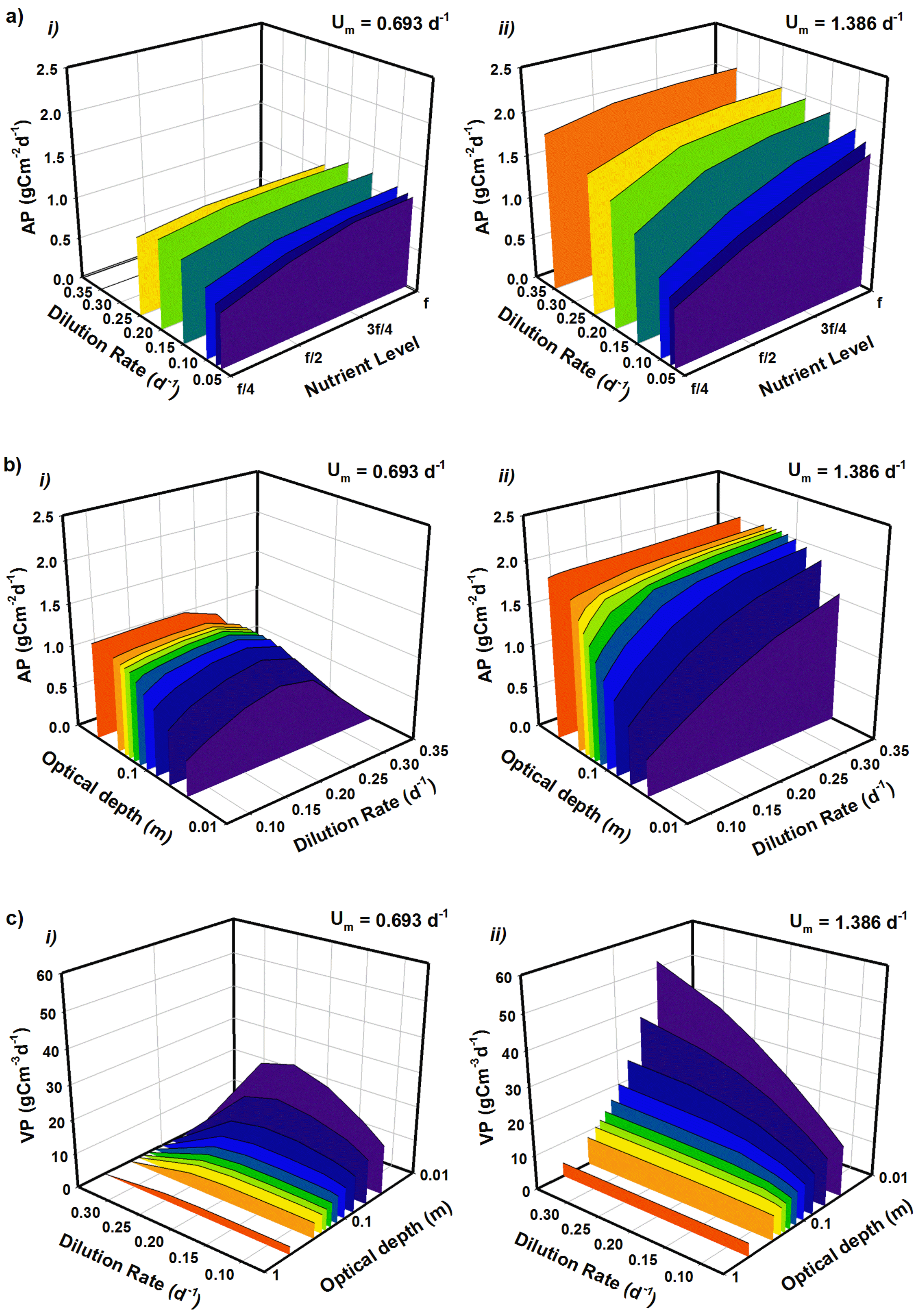
a)
i)
U_m = 0.693 d^-1
AP (gCm^-2 d^-1)
2.5
2.0
1.5
1.0
0.5
0.0
0.35
0.30
0.25
0.20
0.15
0.10
0.05
Dilution Rate (d^-1)
f/4
f/2
3f/4
f
Nutrient Level
ii)
U_m = 1.386 d^-1
AP (gCm^-2 d^-1)
Dilution Rate (d^-1)
Nutrient Level
b)
i)
U_m = 0.693 d^-1
AP (gCm^-2 d^-1)
Optical depth (m)
0.1
0.01
0.10
0.15
0.20
0.25
0.30
0.35
Dilution Rate (d^-1)
ii)
U_m = 1.386 d^-1
AP (gCm^-2 d^-1)
Optical depth (m)
Dilution Rate (d^-1)
c)
i)
U_m = 0.693 d^-1
VP (gCm^-3 d^-1)
60
50
40
30
20
10
0
0.30
0.25
0.20
0.15
0.10
Dilution Rate (d^-1)
1
0.1
0.01
Optical depth (m)
ii)
U_m = 1.386 d^-1
VP (gCm^-3 d^-1)
Dilution Rate (d^-1)
Optical depth (m)

Figure S2

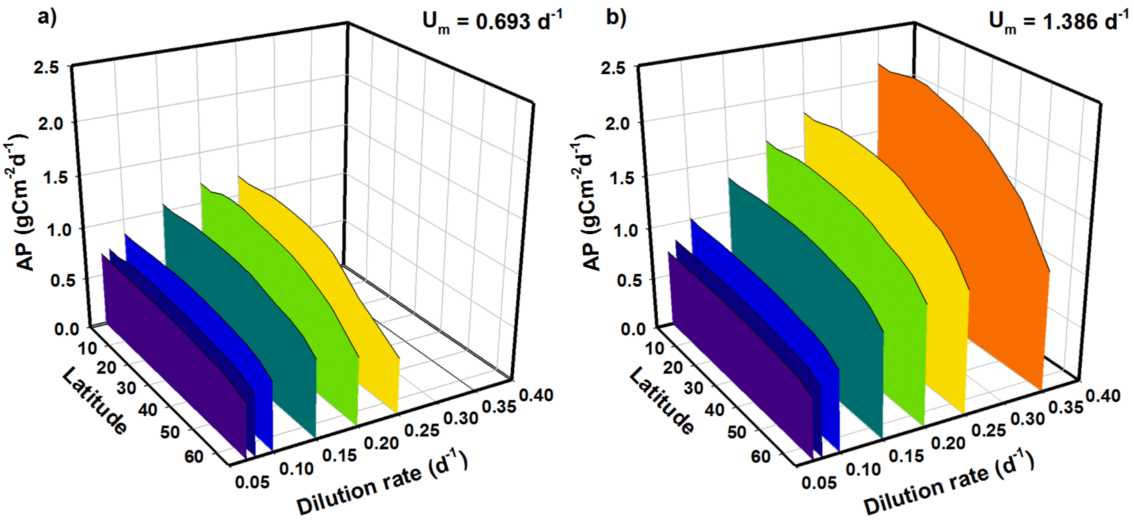
a)
U$_m$ = 0.693 d^{-1}
AP (gCm^{-2}d^{-1})
Latitude
Dilution rate (d^{-1})
b)
U$_m$ = 1.386 d^{-1}
AP (gCm^{-2}d^{-1})
Latitude
Dilution rate (d^{-1})

Figure S3

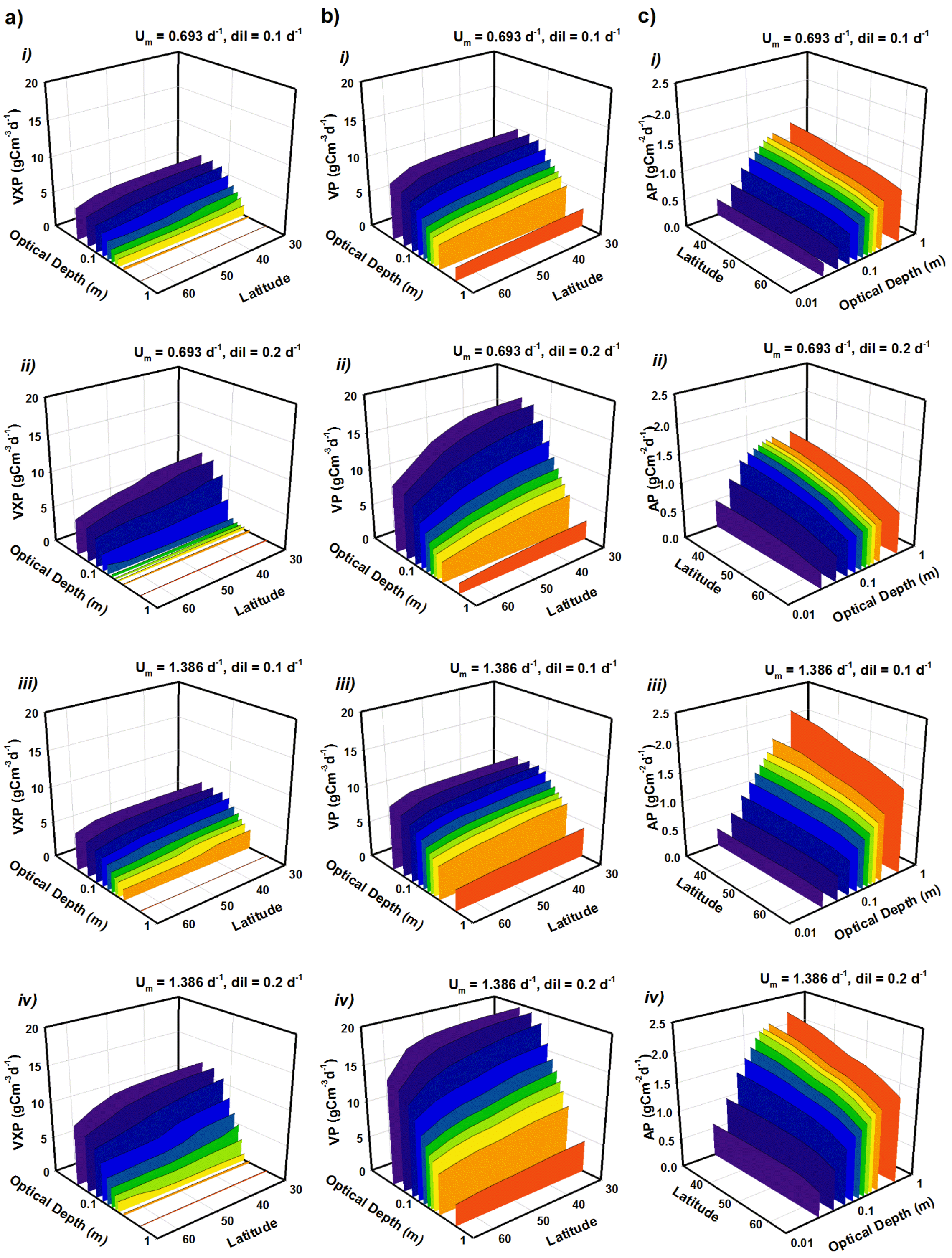
a)
b)
c)
U_m = 0.693 d^-1, dil = 0.1 d^-1
U_m = 0.693 d^-1, dil = 0.2 d^-1
U_m = 1.386 d^-1, dil = 0.1 d^-1
U_m = 1.386 d^-1, dil = 0.2 d^-1
i)
ii)
iii)
iv)
VXP (gCm^-3d^-1)
VP (gCm^-3d^-1)
AP (gCm^-2d^-1)
Optical Depth (m)
Latitude

Figure S4

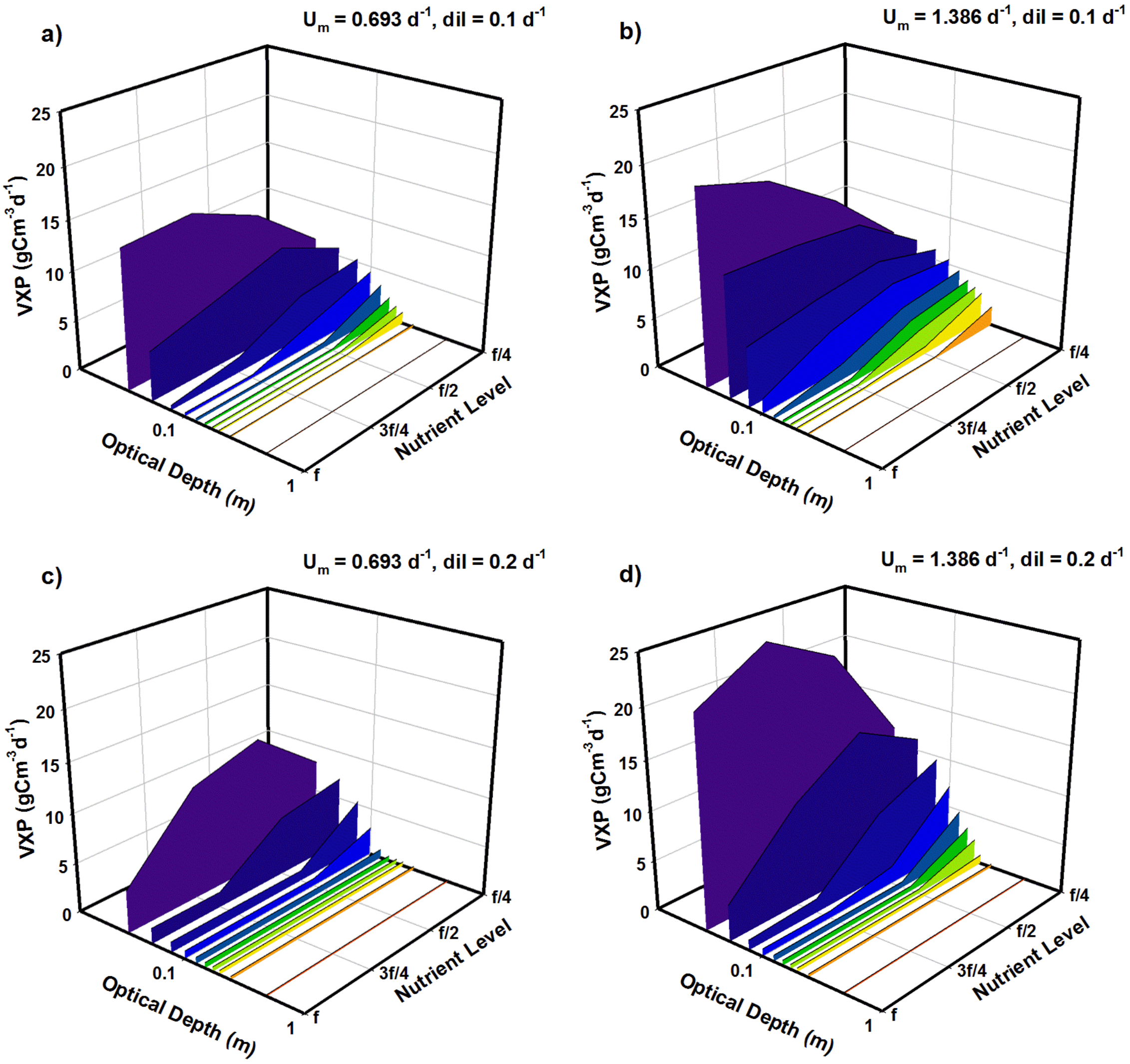

a)
U_m = 0.693 d^{-1}, dil = 0.1 d^{-1}
b)
U_m = 1.386 d^{-1}, dil = 0.1 d^{-1}
c)
U_m = 0.693 d^{-1}, dil = 0.2 d^{-1}
d)
U_m = 1.386 d^{-1}, dil = 0.2 d^{-1}
VXP ($gCm^{-3}d^{-1}$)
0
5
10
15
20
25
Optical Depth (m)
0.1
1
Nutrient Level
f/4
f/2
3f/4
f

Figure S5

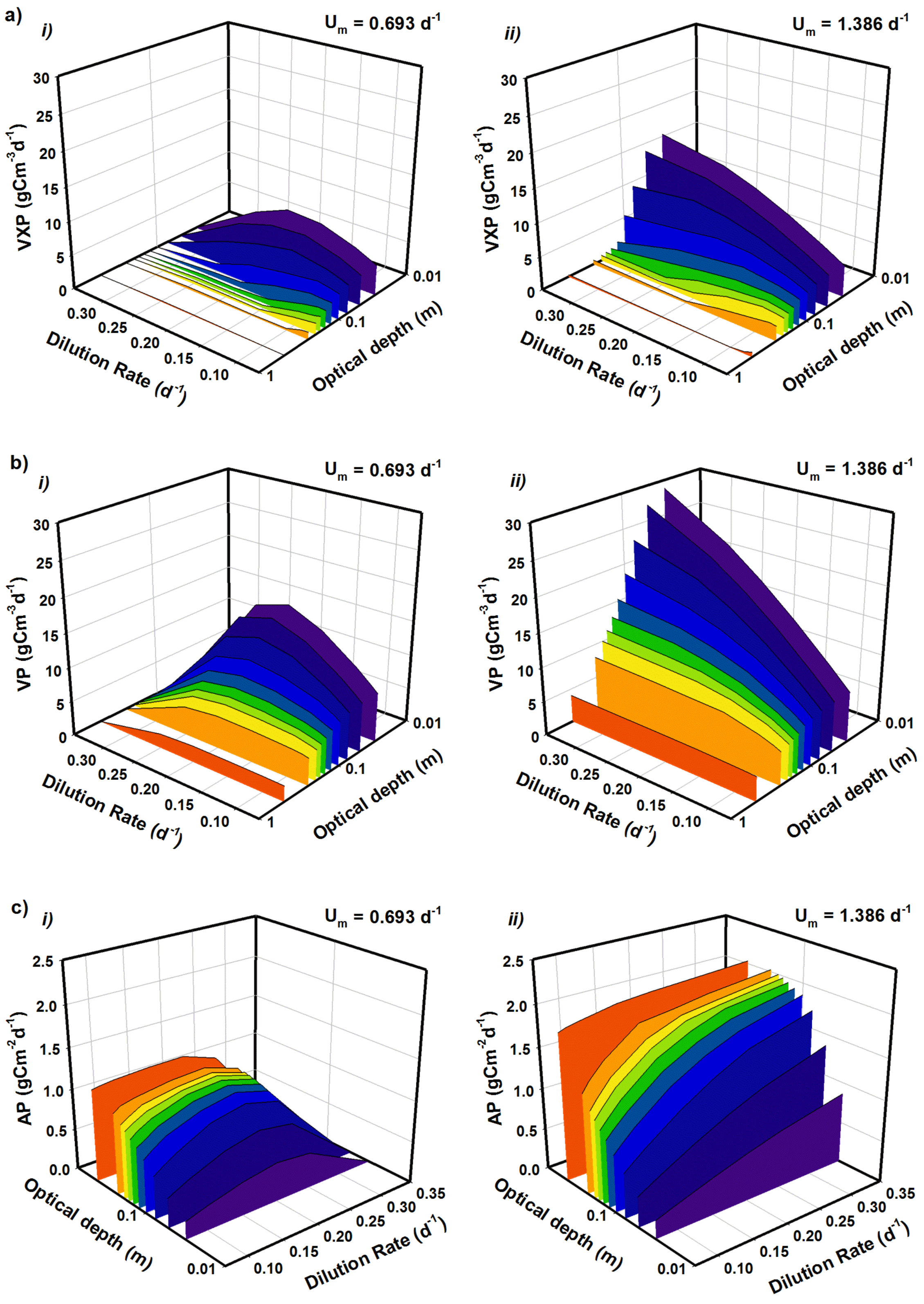
a)
i)
U_m = 0.693 d^{-1}
VXP (gCm^{-3}d^{-1})
Dilution Rate (d^{-1})
Optical depth (m)
ii)
U_m = 1.386 d^{-1}
b)
VP (gCm^{-3}d^{-1})
c)
AP (gCm^{-2}d^{-1})

Figure S6
